# Junctional and allele-specific residues are critical for MERS-CoV neutralization by an exceptionally potent germline-like antibody

**DOI:** 10.1038/ncomms9223

**Published:** 2015-09-15

**Authors:** Tianlei Ying, Ponraj Prabakaran, Lanying Du, Wei Shi, Yang Feng, Yanping Wang, Lingshu Wang, Wei Li, Shibo Jiang, Dimiter S. Dimitrov, Tongqing Zhou

**Affiliations:** 1 Key Laboratory of Medical Molecular Virology of Ministries of Education and Health, Shanghai Medical College, Fudan University, Shanghai, 200032 China; 2 Protein Interactions Section, Cancer and Inflammation Program, Center for Cancer Research, National Cancer Institute, National Institutes of Health, Frederick, 21702 Maryland USA; 3 Lindsley F. Kimball Research Institute, New York Blood Center, New York, 10065 New York USA; 4 Vaccine Research Center, National Institute of Allergy and Infectious Diseases, National Institutes of Health, Bethesda, 20892 Maryland USA; 5 Present Address: Present address: Sanofi Pasteur Biologics, Cambridge, Massachusetts 02139, USA., ,

**Keywords:** Viral infection, Virus structures, Structural biology

## Abstract

The MERS-CoV is an emerging virus, which already infected more than 1,300 humans with high (∼36%) mortality. Here, we show that m336, an exceptionally potent human anti-MERS-CoV antibody, is almost germline with only one somatic mutation in the heavy chain. The structure of Fab m336 in complex with the MERS-CoV receptor-binding domain reveals that its IGHV1-69-derived heavy chain provides more than 85% binding surface and that its epitope almost completely overlaps with the receptor-binding site. Analysis of antibodies from 69 healthy humans suggests an important role of the V(D)J recombination-generated junctional and allele-specific residues for achieving high affinity of binding at such low levels of somatic hypermutation. Our results also have important implications for development of vaccine immunogens based on the newly identified m336 epitope as well as for elucidation of mechanisms of neutralization by m336-like antibodies and their elicitation *in vivo*.

**Supplementary information:**

The online version of this article (doi:10.1038/ncomms9223) contains supplementary material, which is available to authorized users.

## Abstract (web summary)

m336 is an exceptionally potent germline-like antibody against the emerging MERS-CoV virus. Here, the authors solve the structure of m336 in complex with MERS-CoV receptor-binding domain and use it to reveal a role of junctional and allele-specific residues in the interaction and suggest implications for vaccine development.

**Supplementary information:**

The online version of this article (doi:10.1038/ncomms9223) contains supplementary material, which is available to authorized users.

## Graphical abstract

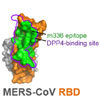

## Introduction

The recent discovery of neutralizing monoclonal antibodies (mAbs) with exceptional potency and breadth against human immunodeficiency virus-1 (HIV-1) and influenza has renewed the interest in designing vaccines to elicit similar antibodies *in vivo*^1,2,3,4^. However, the vaccine development has been impeded by the fact that these broadly neutralizing antibodies (bnAbs) are highly divergent from their putative germline predecessors, and that the designed germline antibodies showed little or no measurable binding to viral antigens^5,6,7^. Interestingly, neutralizing antibodies against some emerging viruses including severe acute respiratory syndrome coronavirus (SARS-CoV), Nipah and Hendra viruses, which cause acute infections, could be elicited within relatively short period of time after infection, and some of these antibodies were very close to their putative germline predecessors^8,9,10^. Therefore, the structural characterization of the interactions between these germline-like antibodies and the viruses may represent a unique opportunity to understand the initial elicitation mechanism of the bnAbs and may eventually help the design of effective vaccine immunogens^4,7,11,12,13,14^.

The Middle East respiratory syndrome coronavirus (MERS-CoV) is a novel, highly pathogenic human coronavirus first recognized in September 2012 in Saudi Arabia in a patient who exhibited SARS-like respiratory syndrome and died from severe pneumonia and renal failure^15,16^. Since September 2012, MERS cases have been reported in more than 20 countries^15,16^, and 1,356 laboratory-confirmed cases have been reported to the World Health Organization as of 26 June 2015, including at least 484 related deaths ( http://www.who.int/csr/don/26-june-2015-mers-korea/en/). Of note, MERS-CoV is phylogenetically distinct from any human coronavirus including SARS-CoV, but more related to the bat coronaviruses HKU4 and HKU5 (refs 17, 18). We and others have found that MERS-CoV most likely originated in bats and has adapted to use human receptor dipeptidyl peptidase-4 (DPP4) to gain entry into host cells^19,20^. Dromedary camels in the Middle East and Africa are considered to be one of the intermediate transmitters of MERS-CoV from bats to humans^21,22,23,24^. Indeed, it has been found that coronaviruses are able to rapidly and stably adapt to new host species^25^. These findings, along with the fact that MERS-CoV emerged only a decade after the appearance of the first highly pathogenic human coronavirus SARS-CoV and has a higher mortality rate (∼36 versus ∼10%), suggest that coronaviruses could be a continuous and long-term threat to human health.

Therefore, we and others have developed human neutralizing mAbs^26,27,28,29^ against the virus for use as candidate therapeutics and templates for vaccine immunogens as well as tools to understand immune responses against this newly emerged virus. The most potent mAb, m336, bound to MERS-CoV receptor-binding domain (RBD) with subnanomolar avidity, and inhibited infection of pseudotyped and live MERS-CoV with an inhibitory concentration required for 50% neutralization (IC_50_) of 0.005 and 0.07 μg ml^−1^, respectively^26^.

In this study, we analysed the immunogenetic origin of m336 and solved its crystal structure in complex with the MERS-CoV RBD. The antibody precisely targets the receptor-binding site (RBS) on MERS-CoV, and a relatively significant number (three) of junctional residues in the heavy-chain complementarity-determining region 3 (HCDR3) and two allele-specific IGHV1-69*06 germline-encoded residues are critical for the RBD binding. Germline mAbs typically exhibit excellent drugability properties, especially lower immunogenicity^11^, and therefore m336-like mAbs may not only be effective templates for development of vaccine immunogens but also could have great potential for prophylaxis and therapy of MERS-CoV infection in humans.

## Results

### Crystal structure of MERS-CoV RBD in complex with Fab m336

To elucidate the molecular mechanisms of MERS-CoV neutralization by m336 and identify possible templates for development of vaccine immunogens, we determined the crystal structure of its complex with the virus RBD. We robotically screened crystallization conditions for an endoglycosidase H-treated MERS-CoV RBD complexed with the Fab m336. Crystals of diffraction quality were obtained from a condition containing 20% mono-Methyl polyethylene glycol 2000 (PEG2000 MME). A 2.65-Å resolution data set in space group P2_1_2_1_2_1_ was collected at 22-ID beamline at the Advanced Photon Source with 20% glycerol as cryoprotectant, and structure solution by molecular replacement with the DDP4-bound MERS-CoV RBD and Fab revealed two Fab m336-MERS-CoV RBD complexes occupying an asymmetric unit. Iterative refinement and model building resulted in a structure with R_work_/R_free_ of 19.7%/25.1% (Table 1; Supplementary Fig. 1a).

**Table 1:** Data collection and structure refinement statistics.

	m336-MERS-CoV RBD
*Data collection*	
Wavelength (Å)	1.0000
Space group	P 2_1_ 2_1_ 2_1_
Unit cell	
a, b, c (Å)	47.8, 146.9, 200.5
Resolution (Å)*	50–2.65 (2.74–2.65)
R_merge_ (%)	11.0 (41.0)
R_pim_ (%)	5.8 (23.4)
I/σI	14.0 (2.0)
Completeness (%)	97.3 (90.3)
Redundancy	3.9 (3.2)
*Refinement*	
Resolution (Å)	45.45–2.65 (2.74–2.65)
Unique reflections	41,125 (3,734)
R_work_/R_free_ (%)	19.7 (27.3)/25.1 (35.8)
Number of atoms	
Protein	9,745
Ligands	68
Water	236
Wilson B-factor (Å^2^)	49.1
B-factors (Å^2^)	
Protein	63.0
Ligands	68.3
Solvent	46.3
r.m.s.d.	
Bond lengths (Å)	0.002
Bond angles (^o^)	0.95
Ramachandran	
Favored (%)	95.0
Outliers (%)	0.2
Clashscore	4.0

^*^Statistics for the highest-resolution shell are shown in parentheses.

### Fab m336 targets the MERS-CoV RBS

**Figure 1:**
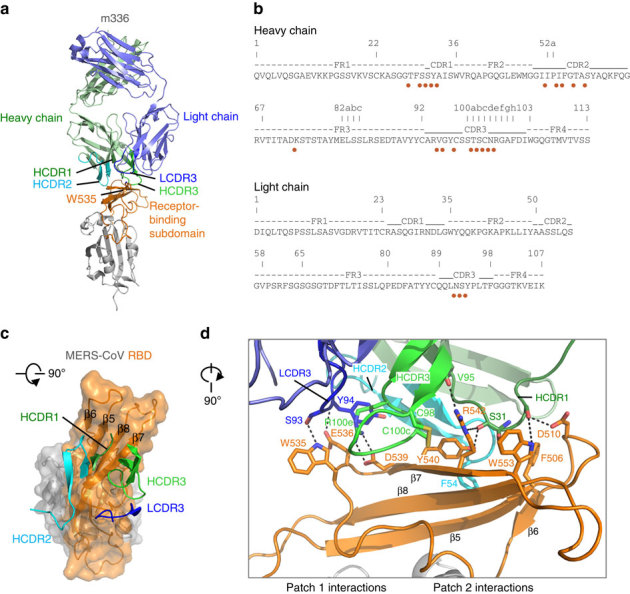
Crystal structures of Fab m336 in complex with MERS-CoV RBD. (**a**) Complex structure in cartoon representation. Heavy and light chains of m336 are coloured light green and light blue, respectively. The receptor-binding subdomain on MERS-CoV RBD is coloured orange with Trp535 shown in shticks representation. (**b**) Sequences of m336 heavy chain and light chain with residues that interact with the MERS-CoV RBD highlighted with orange dots. Antibody residues are numbered according to the Kabat nomenclature. (**c**) Interaction between CDRs of m336 and the MERS-CoV RBD. m336 CDRs are shown in cartoon representation and the MERS-CoV RBD is depicted in semitransparent surface and cartoon. (**d**) Detailed interactions between m336 and patches 1 and 2 of the MERS-CoV RBD. Key residues are highlighted in stick representation. Hydrogen bonds and salt bridges are displayed with dotted lines connecting interacting atoms. The non-canonical disulphide bond between HCDR3 residues Cys98 and Cys100c is in yellow sticks. Both panels (**c**,**d**) are shown as different 90° views from panel (**a**).

Fab m336 interacts with the RBS on the MERS-CoV RBD (Fig. 1a). The interaction surface buries a total area of about 1,700 Å^2^, with 860 Å^2^ contributed by m336 and 820 Å^2^ by MERS-CoV RBD. Even though both the heavy-chain variable domain (V_H_) and the light-chain variable domain (V_L_) of m336 are involved in the interaction, the V_H_ provides >85% binding surface. Within the V_H_, HCDR1, HCDR2 and framework region 3 (FR3) provide ∼58% of the total MERS-CoV RBD-binding surface while HCDR3 (of the V(D)J recombination product) provides ∼29% (Fig. 1b; Supplementary Table 1). With HCDR3 and LCDR3 on one side and HCDR1 and HCDR2 on the other side, the antigen-binding region of m336 forms a claw-shaped surface. The claw grips the RBS with HCDR1 and HCDR2 penetrating into the concave surface formed by β5–β8 and HCDR3 and LCDR3 interacting with the outer edge of β7 and its entrance loop (Fig. 1c). It is of note that a non-canonical disulphide bond forms between Cys98 and Cys100c (the Kabat numbering definition) to stabilize the HCDR3 into a ‘twisted’ loop conformation (Fig. 1d). Similar to the receptor DPP4, m336 interacts with both patches 1 and 2 of the RBS (Fig. 1d).

### Similarity in the RBD recognition by m336 and DPP4

**Figure 2:**
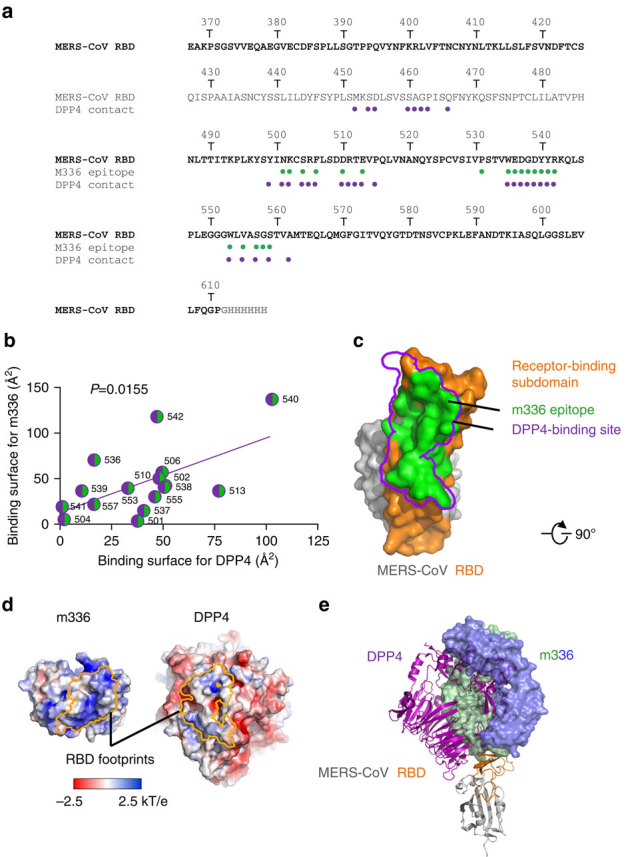
m336 precisely targets the DPP4-binding site on MERS-CoV RBD. (**a**) Mapping of the m336 epitope and DPP4-binding site on MERS-CoV RBD sequence. Residues interacting with DPP4 and m336 are marked with purple and green dots, respectively. MERS-CoV residue numbers are labelled above the sequence. (**b**) Plot of buried surface areas of MERS-CoV RBD residues that interact with both m336 and DPP4. Residue numbers of MERS-CoV RBD residues are labelled for each data point. (**c**) Epitope of m336 (green) overlaps with DPP4-binding site (purple) on the MERS-CoV RBD (orange). (**d**) Comparison of the electrostatic potential surfaces of the MERS-CoV RBD-contacting regions on m336 and DPP4. Molecular surfaces are coloured red for acidic areas and blue for basic areas with the RBD footprints highlighted in orange and olive boundary lines. (**e**) When superposed on MERS-CoV RBD, the volume of m336 overlaps with that of RBD-bound DPP4 indicating antibody m336 exploits similar angle of approach as receptor DPP4 to engage the MERS-CoV and achieves neutralization by blocking DPP4 binding to MERS-CoV.

The m336 epitope is composed of residues from the MERS-CoV RBD RBS, including strands β5–β8 and the loop leading to β7 (Fig. 2a), which overlaps extensively with the DPP4-binding site. Pairwise comparison of ligand-binding surface areas of RBS residues either in the m336 epitope or in the DDP4-binding site^30,31^ showed significant correlation (*P*=0.025), indicating shared usage of residues for binding m336 and receptor DPP4. For RBS residues that interact with both m336 and DPP4, their pairwise surface areas buried by m336 and DPP4 showed even stronger correlation (*P*=0.016; Fig. 2b). Indeed, ∼90% of the m336 epitope surface overlaps with the DPP4-binding site on RBS (Fig. 2c; Supplementary Fig. 1). Interestingly, Trp535_RBD_, which interacts with glycans in DPP4, was engaged by m336 HCDR3 residue Thr100a and LCDR3 residues Asn92 and Ser93. Importantly, in addition to precise targeting of the RBS, critical chemical interactions such as the salt bridges between Asp539_RBD_ and Lys267_DPP4_ were mimicked by HCDR3 Arg100e of m336 (Fig. 1d; Supplementary Table 2). The RBD-binding surfaces on both m336 and DPP4 showed basic electrostatic potentials (Fig. 2d).

Superposition of the RBD in its m336-bound form with that in the DPP4-bound form^30^ resulted in Cα root mean squared deviation (r.m.s.d.) of 0.82 Å, indicating that binding of m336 did not induce significant conformational changes in MERS-CoV RBD. It is interesting to note that the similarity in conformation at the antibody epitope was even higher than the rest of the MERS-CoV RBD (Cα r.m.s.d.=0.50 Å). Such superposition of the MERS-CoV RBD also brought m336 and DPP4 into overlapping positions, with >50% of the Fab m336 encompassed by the volume of DPP4 (Fig. 2e; Supplementary Fig. 2), indicating that m336 and DPP4 use similar angles of approach to target the MERS-CoV RBD.

### Immunogenetic analysis of m336

**Figure 3:**
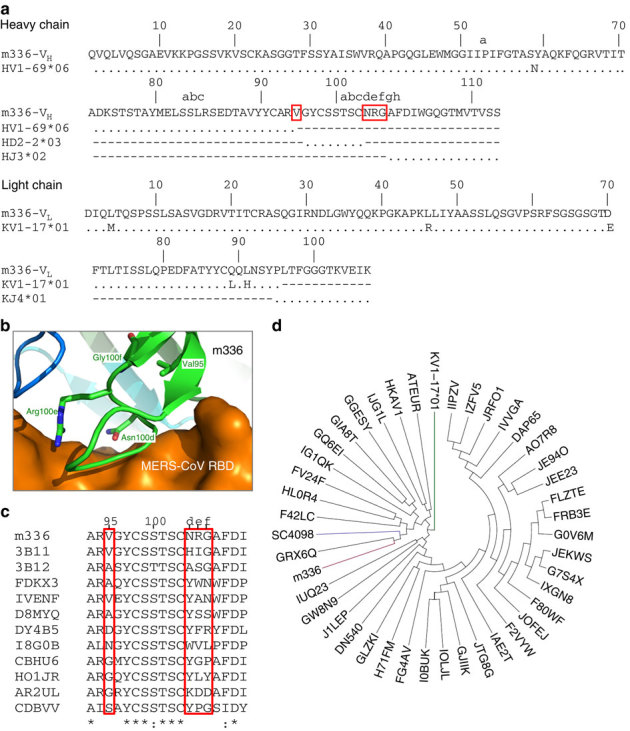
Immunogenetic analysis showed a germline heavy chain with a unique junctional motif. (**a**) m336 V_H_, V_L_ sequence alignments with germline gene segments are shown. Identical residues are denoted by dots and junction residues at HCDR3 are boxed. Antibody residues are numbered according to the Kabat nomenclature. (**b**) Close-up view of interactions between the junctional amino acids in the HCDR3 of m336 with MERS-CoV RBD. (**c**) Amino acid sequences of HCDR3s that were found similar to that of m336 from 454 sequencing analysis of IgM libraries derived from 69 healthy human subjects (denoted by five-letter codes), and from the report by Tang *et al.*^28^ (3B11 and 3B12). Red boxes show the junction amino acid residues in HCDR3s. (**d**) Germline-rooted circular phylogenetic tree of m336 V_L_-like antibody sequences (denoted by five-letter codes, m336 in red and KV1-17*01 in green) found in IgM libraries derived from 69 healthy human subjects and two newborn babies along with the anti-rabies antibody light chain (SC4098, in purple) from the report by Kramer *et al.*^38^.

We performed immunogenetic analysis of m336 using the IMGT tool^32^ to determine the closest V_H_ and V_L_ germline genes and junctional amino acids of V_H_ (Fig. 3a). We found that the m336 V_H_ gene is derived from the IGHV1-69*06 and that the V_L_ gene is from IGKV1-17. Remarkably, the V_H_ gene was almost germline except for a point mutation (one non-silent nucleotide mutation) leading to a change from asparagine (Asn) to serine (Ser) at position 58. m336 was found to be derived from *06 allele that has Phe54 in HCDR2 and Lys73 in FR3. These phenylalanine (Phe) and lysine (Lys) residues are naturally encoded in most IGHV1-69 alleles, which were either found together or alone in alleles *01, *02, *03, *04, *05, *06, *07, *08, *09, *10, *12, *13 and *14. Interestingly, the IGHV1-69 gene was dominantly used in a number of anti-viral antibodies including those targeting HIV-1 gp120 CD4i epitope^33^, influenza HA^4,13,34^, SARS-CoV RBD and henipaviruses^35^. For m336, both allele-specific residues, Phe54 and Lys73, of the IGHV1-69*06, HCDR3 comprising IGHD2-2*03 and IGHJ3*02, and germline HCDR2 contributed to the paratope of V_H_ (Fig. 1b,d). Importantly, genetic and structural analyses further showed that the m336 V_H_ has specific junctional residues at positions 95, 100d, 100e and 100f, which were encoded as amino acids valine (Val), Asn, arginine (Arg) and glycine (Gly), respectively (Fig. 3a) and play a dominant role in HCDR3 as elucidated in the crystal structure (Fig. 3b). Notably, m336 has no somatic mutations in the IGHD and IGHJ genes, which can be unambiguously identified by IMGT/V-Quest (Supplementary Table 3). In contrast, m336 V_L_, composed of IGKV1-17*01 and IGKJ4*01, was found to have five amino acid residue changes (five non-silent and three silent nucleotide mutations) resulting from mutations in the IGKV gene (Fig. 3a; Supplementary Table 4). However, none of the V_L_ somatic mutations was found to be directly involved in binding to RBD, but it is possible that they could influence V_H_–V_L_ interactions or result in other allosteric effects. Further, the detailed structural analysis of m336 complex structure revealed that the CDRH3 residues, particularly, the junctional residues Asn100d and Arg100e, are involved in hydrogen bonding with Gly538 and Asp539 of RBD, respectively, which mimicked the conserved DPP4 interactions Gln286 and Lys267 (Fig. 2a,b; Supplementary Table 2). Also, the junctional residue Arg100e formed a salt bridge with Asp539, which resembled the conserved slat bridge involving Lys267 of DPP4 (Fig. 1d,b; Supplementary Table 2). In summary, we show that the germline-encoded residues, Phe54 in HCDR2 and Lys73 in FR3, which are conserved in many IGHV1-69 alleles, and, importantly, junctional residues of HCDR3 that make conserved interactions that of DPP4, could play critical roles in the m336 paratope interactions.

These findings suggest that the selection of specific junctional amino acids with certain V(D)J recombination and allele-specific amino acids in the heavy chain, and subsequent somatic mutations in the light chain could lead to the high affinity of m336 for its target RBD. However, the adaptability of somatic mutations might depend on the nature of junctional residues that occur far ahead of any somatic mutations and determine the pathway of affinity maturation^36^.

To further investigate potential immunogenetic mechanisms such as IGHV1-69 recombination frequency with specific IGHD and IGHJ genes among other germlines and conserved amino acid residues at allelic and junctional positions, we analysed in detail the deep sequencing data of our antibodyome studies from 69 healthy human donors^35^. Our analysis revealed that a significant (12.7%) proportion of expressed V_H_ genes were productively recombined from the IGHV1-69 gene (Supplementary Fig. 3). Among a total of 9,454 sequences of IGHV1-69 germline origin, 6,512 sequences had the germline-encoded Phe54 and 3,057 sequences had the germline-encoded Lys73 natural allelic residues in the population of 69 individuals, while 1,452 sequences (15.4%) had both residues, Phe54 and Lys73, such as found in m336. This suggests a potential mechanism contributing to individually variable but effective elicitation of m336-like neutralizing antibodies in response to MERS-CoV infection. We further explored the V_H_ sequences from our 454 antibody sequencing database to find V_H_-related m336-like antibodies. We found nine m336-related V_H_ sequences from 69 volunteers that shared the same IGHV1-69 germline family with a characteristic disulphide bond at the middle of HCDR3 (Fig. 3c). The commonalities among the HCDR3s included the similar HCDR3 length of 18 amino acids and the usage of D2-2 segment. Notably, m336 had a unique junctional motif as compared with those nine V_H_ sequences as well as to those two anti-MERS-CoV antibodies (3B11 and 3B12) recently reported^28^ (Fig. 3c). A disulphide bond characterized by the IGHD2-2*03 gene was conserved in all the HCDR3s, implying the same IGHD reading frame usage in all those nine V_H_ sequences. The disulphide-bonded cysteine residues in the HCDR3 of m336 involved in the direct binding to MERS-CoV RBD (Fig. 1b,d) suggested a possible functional role of this HCDR3 disulphide motif. Compared with the other anti-MERS-CoV antibodies, 3B11 and 3B12, m336 used a different V_L_ derived from IGK1-17, whereas 3B11 and 3B12 used IGKV3-20 and IGKV4-1, respectively for their V_L_ sequences.

Although antibody phage display technique has proved to be an effective method for generating mAbs, the mAbs so obtained from random combinations of V_H_ and V_L_ may not reflect the natural V_H_/V_L_ pairing as found in immune repertoire. To assess the potential compatibility of V_H_/V_L_ pairing in m336, we searched through the IMGT/3Dstructure-DB. We found that the same m336-specific V_H_/V_L_ pairing, IGHV1-69/IGK1-17, exists in the dengue cross-reactive hybridoma-derived mAb 2H12 and anti-Homo sapiens epidermal growth factor receptor (EGFR) antibody, imgatuzumab, indicating the immunologically relevant cognate V_H_/V_L_ pairing type for m336.

To examine the role of IGKV1-17 usage in m336 V_L_, we further analysed the IGKV1-17 lineage sequences retrieved from naive IgM repertoires of 69 healthy human subjects as well as in neonatal IgM repertoires of two newborn babies^37^. We found 40 unique m336 V_L_-like sequences with the IGKV1-17 lineage that shared some pre-existing amino acid mutations with m336 V_L_ (Supplementary Fig. 4). Phylogenetic tree analysis of the m336 V_L_-like sequences revealed a highly germline-related nature of the m336 V_L_ (Fig. 3d). We previously found that ∼70% of the heavy and light chains had one to five amino acid changes as naturally occurring somatic hypermutation in the cord blood IgM repertoire^37^. An interesting aspect of our study is that most of the m336 V_L_ mutations are also found as pre-existing mutations associated with certain IGKV1-17 lineage sequences in those IgM repertoires (Supplementary Fig. 4). Further, we identified an anti-rabies virus antibody SC4098 by BLAST search against the GenBank database, whose V_L_ chain has all the four mutations identical to those in m336 V_L_ as compared with the IGKV1-17 germline^38^. This implies a possible functional role of these V_L_ mutations. Accordingly, these V_L_ mutations, although in a lesser extent, along with the unique junctional residues and allele encoding residues of V_H_, might have played a role in the high-affinity binding to MERS-CoV.

Taken together, these results suggest that m336 V_H_ uses the key immunogenetic elements including naturally occurring Phe54 and Lys73-encoded allelic-specific amino acids and specific junctional amino acid residues while m336 V_L_ relies on somatic mutations for deriving its high affinity in an unprecedented manner.

### Binding and neutralization activity of germline precursors

**Figure 4:**
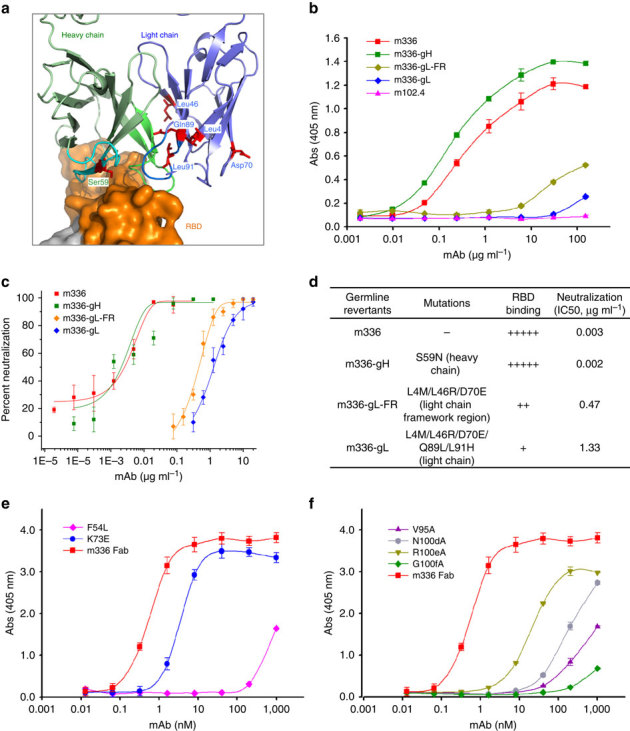
The binding and neutralization activities. (**a**) Representation of the amino acids in m336 that are divergent from those in the predicted germline precursor. None of the five residues in the light chain has any direct contact with the MERS-CoV RBD. (**b**) Binding of IgG1s m336, m336-gH, m336-gL-FR, m336-gL and a negative control IgG1 m102.4 to MERS-CoV S1 protein measured by ELISA. (**c**) Neutralization of viruses pseudotyped with the MERS-CoV S glycoprotein. (**d**) A summary of the binding and neutralization results of m336 and the germline revertants. (**e**) Binding of Fab m336 allele-specific mutants (F54L and K73E) to MERS-CoV S1 protein measured by ELISA. (**f**) Binding of Fab m336 junctional mutants (V95A, N100dA, R100eA and G100fA) to MERS-CoV S1 protein measured by ELISA.

It was surprising that m336 as an exceptionally potent MERS-CoV neutralizer had only six amino acid changes from its inferred germline precursor. To determine whether these mutations are essential for the potency of m336, we reverted the residue(s) in the heavy chain (m336-gH), light chain (m336-gL) or light-chain framework region (m336-gL-FR) to their germline counterparts, respectively (Fig. 4). The binding of m336 and the germline predecessors to the recombinant MERS-CoV S1 protein was evaluated by enzyme-linked immunosorbent assay (ELISA; Fig. 4b,d) and Biacore (Supplementary Fig. 5), and their neutralization activity against MERS-CoV S1-pseudotyped viruses in Huh-7 cells was determined using methods previously described^26^ (Fig. 4c,d). The reversion of the only mutation in the heavy chain (S59N) did not have any effect on the MERS-CoV-binding affinity and neutralizing activity of m336. The m336-gH exhibited potent neutralizing activity with an IC_50_ of 0.002 μg ml^−1^, which is very similar to that of m336 measured here (0.003 μg ml^−1^) or described previously (0.005 μg ml^−1^).

The m336 light chain contains three somatic mutations in the framework region (Leu4, Leu46 and Asp70) and two in the LCDR3 region (Gln89 and Leu91). None of these residues makes direct contact with the MERS-CoV RBD. Interestingly, reversion of the three framework somatic mutations of m336 to their germline counterparts (m336-gL-FR) not only decreased the binding to MERS-CoV RBD (Fig. 4b) but also markedly reduced the neutralization potency (Fig. 4c). The neutralization activity of m336-gL-FR (IC_50_=0.47 μg ml^−1^) was two orders of magnitude lower than that of m336 (IC_50_=0.003 μg ml^−1^). Complete reversion of somatic mutations in the m336 V_L_ further decreased RBD-binding activity and MERS-CoV-neutralizing activity. Interestingly, the dissociation rate constants of the four antibodies are similar but the association rate constants of m336 and m336-gH are much higher than that of m336-gL and m336-gL-FR (Supplementary Fig. 5). The IC_50_ value of m336-gL was 1.33 μg ml^−1^, threefold higher than that of m336-gL-FR. Consistent with the immunogenetic analysis, these results support the importance of naturally occurring V_L_ mutations, and demonstrate that non-contact residues in the m336 light chain are essential for its binding and potency.

### Binding activity of junctional and allele-specific mutants

To further demonstrate the importance of junctional and allele-specific residues in the binding of m336 to RBD, we generated a series of m336 mutations and measured their binding to MERS-CoV S1 protein by ELISA (Fig. 4e,f). m336 was derived from IGHV1-69*06 allele that has Phe54 in HCDR2 and Lys73 in FR3. In the 14 IGHV1-69 alleles, eight of them have a phenylalanine at position 54 while the other six alleles (*02, *04, *08, *09, *10 and *11) have a leucine. Interestingly, the m336 F54L mutant was found to have greatly reduced binding affinity to MERS-CoV S1 protein with at least 1,000-fold reduced EC50 as compared with the wild-type m336 (Fig. 4e). Similarly, half of the 14 alleles have a lysine at position 73 while the other 7 alleles (*01, *03, *05, *07, *11, *12 and *13) have a glutamic acid, and the m336 K73E mutant was found to have at least 10-fold reduced MERS-CoV S1 binding affinity as compared with m336. The alanine mutations of the four junctional amino acids (V95A, N100dA, R100eA and G100fA) also showed severely reduced binding to MERS-CoV S1 as compared with the wild-type m336 (Fig. 4f). These results, along with the structural analysis, confirmed that the junctional and allele-specific amino acids in m336 are critical for its MERS-CoV RBD binding and virus neutralization.

## Discussion

Elicitation of highly somatically mutated bnAbs against viruses with high antigenic diversity, such as HIV, has been challenging when traditional vaccine approaches are applied. Thus, an alternative vaccination strategy has been proposed to first activate naive mature B cells expressing unmutated germline B cell receptors with germline antibody-binding immunogens, followed by immunogen binding to intermediate antibodies to guide the immune system through the complex maturation pathways, resulting in, finally, the elicitation of those antibodies with specific sequences^11^.

However, it appears that potent antibodies with broad neutralizing activity against some viruses, in this case the MERS-CoV, can naturally exist with very low level of somatic hypermutation. The MERS-CoV RBD is highly conserved with very few mutations. By analysing the known RBD sequences, we found that the epitope of m336 does not overlap with any of the point mutations (Supplementary Fig. 6). By using genetic and structural analyses, we discovered that a germline-encoded antibody, m336, could achieve high-affinity recognition and neutralization of the newly emerged MERS-CoV, mostly by using allele-specific residues, as well as junctional amino acids. It is noteworthy that even though germline sequences of specific antibodies (for example, HIV-1 bnAbs) can be predicted by reconstructing B cell clonal lineages^39^, it may not be possible to infer the exact naturally occurring junctional amino acids, which were introduced by V(D)J rearrangement, a critical process preceding somatic hypermutation. Therefore, the junctional amino acids of the mature antibodies are usually retained or bioinformatically derived in the design of germline-encoded precursor antibodies^5,6,7,40^. Notably, it has been demonstrated that adaptability of somatic mutations might depend on the nature of junctional amino acids that occur far ahead of any somatic mutations and could determine the maturation pathway of antibodies^36^. This finding, coupled with the fact that the matured antibodies for some viruses, for example, HIV-1, are highly somatically mutated, suggests that the junctional residues in matured antibodies may be quite different from their initial germline counterparts, a phenomenon that could have important consequences for the elicitation of bnAbs. Therefore, we propose here that alternative strategies to identify naturally occurring germline predecessors of bnAbs should be explored through human antibodyome rather than theoretically reconstructing them, thereby confounding the rational design of otherwise effective vaccine immunogens.

Interestingly, we also found that m336 has unique junctional amino acids in the HCDR3, as compared with the related V_Hs_ identified by deep sequencing of antibodyomes from 69 healthy humans, as well as other recently identified anti-MERS-CoV antibodies of IGHV1-69 origin^28,35^. These junctional amino acids play an important role as they are directly involved in the interaction between m336 and MERS-CoV RBD. Particularly, all the junctional amino acids in m336 participate in antigen recognition, and some, for example, Arg100e that forms salt bridges with Asp539 of MERS-CoV RBD, are critical for the high-affinity interaction. Other key immunogenetic elements including naturally occurring Phe54 and Lys73-encoded allelic-specific amino acids have also been identified to play a role in the high-affinity binding of m336 to MERS-CoV RBD. Our results suggest that the direct antigen recognition effects of the junctional residues in the HCDR3, which could affect subsequent somatic hypermutation and the maturation pathway of an antibody^36^, should be considered in the design of germline-encoded precursor antibodies and related vaccine strategies. These results also demonstrate that junctional diversity and specific alleles can potentially contribute to individual differences in humoral immune responses, and, as such, they can be key cofactors in selecting potent neutralizing antibodies.

The identification of germline-like potent MERS-CoV-neutralizing antibodies implies that elicitation of m336-like neutralizing antibody response by immunization could be relatively quick and effective. The crystal structure of the MERS-CoV RBD in complex with the m336 Fab also shows that m336 bound to an area on the RBS of the MERS-CoV RBD that encompasses both patch 1 and patch 2 residues. With its epitope almost completely overlapping the binding site of the natural receptor DPP4, m336 not only mimicked critical interactions between DDP4 and MERS-CoV RBD^30,31^ but also exploited an angle of approach for recognition similar to that of DPP4 to achieve potent neutralization. It is reasonable to speculate that the development of viral escape mutants to m336 should be unfavourable, since the reduced binding to m336 of the mutant viruses could also result in the reduced binding to the cellular receptor DPP4. Thus, the m336 epitope on the RBD identified here may aid in the development of highly effective MERS-CoV vaccines and the antibody itself, due to its germline nature, could have great potential for prophylaxis and therapy of MERS-CoV infection in humans.

## Methods

### Protein expression and purification

MERS-CoV *Eng1* RBD with a C-terminal HRV-3c cleavage site and His6x purification tag was expressed in GnTi^−/−^ cells^41^. The MERS-CoV RBD protein was purified by nickel-nitrilotriacetic acid (Ni-NTA) affinity chromatography followed by size exclusion chromatography using 1 × PBS as buffer. The m336-gH mutant was generated using QuikChange II XL site-directed mutagenesis kit (Stratagene) with the m336 IgG1-expressing plasmid as a template; the m336 F54L, K73E, V95A, N100dA, R100eA and G100fA mutants were generated with the m336 Fab-expressing plasmid as a template. The light chains of m336-gL-FR and m336-gL were synthesized by GenScript (Piscataway, NJ) and inserted into the m336 IgG1-expressing plasmid, respectively, to replace the original light chain of m336. The antibodies were expressed and analysed, and protein purity was estimated as >95% by SDS–polyacrylamide gel electrophoresis and protein concentration was measured spectrophotometrically (NanoVue, GE Healthcare).

### Crystallization and data collection

The antigen-binding fragment of antibody m336 was prepared using Lys-C (Roche) digestion with an IgG/Lys-C ratio of 4,000:1 (w/w)^2^. The RBD protein was then mixed with the m336 Fab in a 1:1.5 molar ratio and incubated for 30 min at room temperature. The complexes were purified by size exclusion chromatography (Superdex S200; GE Healthcare) and concentrated to ∼8 mg ml^−1^ for crystallization screening.

Initial crystallizations were carried out at 20 °C using a Mosquito crystallization robot (TTP Labtech, UK) and commercially available Hampton (Hampton Research), Precipitant Synergy (Emerald Biosystems) and Wizard (Emerald Biosystems) crystallization screens. Droplets were allowed to equilibrate at 20 °C and imaged at scheduled times with Rock Imager (Formulatrix, MA). Robotic crystal hits were optimized manually using the hanging drop vapor-diffusion method and crystals of diffraction quality were obtained by mixing 0.5 μl of protein complex and 0.5 μl of reservoir solutions containing 20% mono-Methyl polyethylene glycol 2000 and 100 mM HEPES, pH 7.5.

Diffraction data of the m336/RBD crystals were collected under cryogenic conditions with a buffer containing 20% mono-Methyl polyethylene glycol 2000 and 100 mM HEPES, pH 7.5 and 20% glycerol as cryoprotectant, at beamline ID-22 (SER-CAT) at the Advanced Photon Source, Argonne National Laboratory, with 1.0000 Å radiation. The 2.65 Å resolution data set was processed and scaled with HKL2000 (ref. 42) in P2_1_2_1_2_1_ space group.

### Structure determination and refinement

The structure of the m336:MERS-CoV RBD complex was solved by molecular replacement using Phaser^43^ in the CCP4 Program Suite^44^. To place the two copies of m336/MERS-CoV RBD complex in the asymmetric unit, MERS-CoV RBD from PDB ID 4KQZ was used as the initial model to locate the MERS-CoV protein. CDR-loop-trimmed variable domain of Fab VRC-PG04 (PDB ID 3SE9) and its constant domain were used separately to locate corresponding domains of Fab m336 in the structure.

Refinements were carried out with PHENIX^45^ with a cross validation (R_free_) test set containing 5% of the data. Starting with torsion-angle simulated annealing with slow cooling, iterative manual model building was carried out in COOT^46^ with maps generated from combinations of positional, individual *B*-factor and TLS refinement algorithms. Ordered solvents were added during each macro cycle. Structure validations were performed periodically during the model building/refinement process with MolProbity^47^. Final refinement statistics are summarized in Table 1.

### ELISA

Binding of antibodies to the MERS-CoV S1 protein (Sino Biological Inc.) was measured by ELISA. Serial fivefold dilutions of antibodies starting from 1 μM were diluted in PBS and assayed for binding to MERS-CoV S1 protein. After washing, bound antibodies were detected by horseradish peroxidase (HRP)-conjugated goat anti-human IgG Ab (Sigma-Aldrich, cat. no. A0170) or horseradish peroxidase-conjugated anti-FLAG Ab (Sigma-Aldrich, cat. no. A8592).

### Surface plasmon resonance binding

Surface plasmon resonance measurements were performed using a BIAcore X100 instrument (GE Healthcare). MERS-CoV S1 protein (Sino Biological Inc.) diluted in 10 mM sodium acetate buffer (pH 5.5) was immobilized on a CM5 biosensor chip using a primary amine coupling method. The running buffer was allowed to flow through the cells at a rate of 30 μl min^−1^. The analytes consisted of serial dilution of proteins between 500 to 0.05 nM (500, 50, 5, 0.5 and 0.05 nM).

### Neutralization assays

For measuring the neutralization activities of m336, m336-gH, m336-gL-FR and m336-gL, Huh-7 cells were infected with MERS-CoV-S pseudoviruses in the presence of serial dilutions of antibodies at indicated concentrations. The culture was re-fed with fresh medium 12 h post infection and incubated for an additional 72 h. Cells were washed with PBS and lysed using lysis reagent included in a luciferase kit (Promega). Aliquots of cell lysates were transferred to 96-well flat-bottom luminometer plates (Costar), followed by addition of luciferase substrate (Promega). Relative light units were determined immediately using the Ultra 384 luminometer (Tecan USA).

### Immunogenetic analysis

Antibody cDNA libraries constructed from peripheral blood B cells of 10 and 59 healthy donors^48,49^ were sequenced using the 454 sequencing technology^35^. Informed consent was obtained from all human participants. Details of quality control and 454 high-throughput sequence analysis of IgM antibody variable domains consisting of all three CDRs along with FRs were elaborated elsewhere^37^. Briefly, IMGT/HighV-QUEST^50^ was used for immunogenetics and SAS JMP10 (SAS Institute, Cary, NC) was used for statistical analyses. The output results from the IMGT/HighV-QUEST analysis were stored at a local PostgreSQL database. Structured Query Language (SQL) was used to retrieve the IGHV1-69 and IGKV1-17 lineage sequences for immunogenetic analysis. Sequence alignments were made with ClustalW2. Phylogenetic tree analysis was performed using Archaeopteryx.

## Additional information

**Accession codes:** Atomic coordinates and structure factor amplitudes for the m336-MERS-CoV RBD complex have been deposited in the PBD under accession code 4XAK.

**How to cite this article:** Ying, T. *et al.* Junctional and allele-specific residues are critical for MERS-CoV neutralization by an exceptionally potent germline-like antibody. *Nat. Commun.* **6**:8223 doi: 10.1038/ncomms9223 (2015).

## Supplementary information

Supplementary Information Supplementary Figures 1-6, Supplementary Tables 1-4 (PDF 887 kb)

## Data Availability

Protein Data Bank
4XAK
