# Supplementary material for: Junctional and allele-specific residues are critical for MERS-CoV neutralization by an exceptionally potent germline-like antibody
Source: Nat Commun. 2015 Sep 15;6:8223. doi: 10.1038/ncomms9223 (PMC4571279; doi:10.1038/ncomms9223)
Supplement: Supplementary Information — Supplementary Figures 1-6, Supplementary Tables 1-4 (PDF 887 kb) [file 41467_2015_BFncomms9223_MOESM1267_ESM.pdf]

Supplementary Figure 1

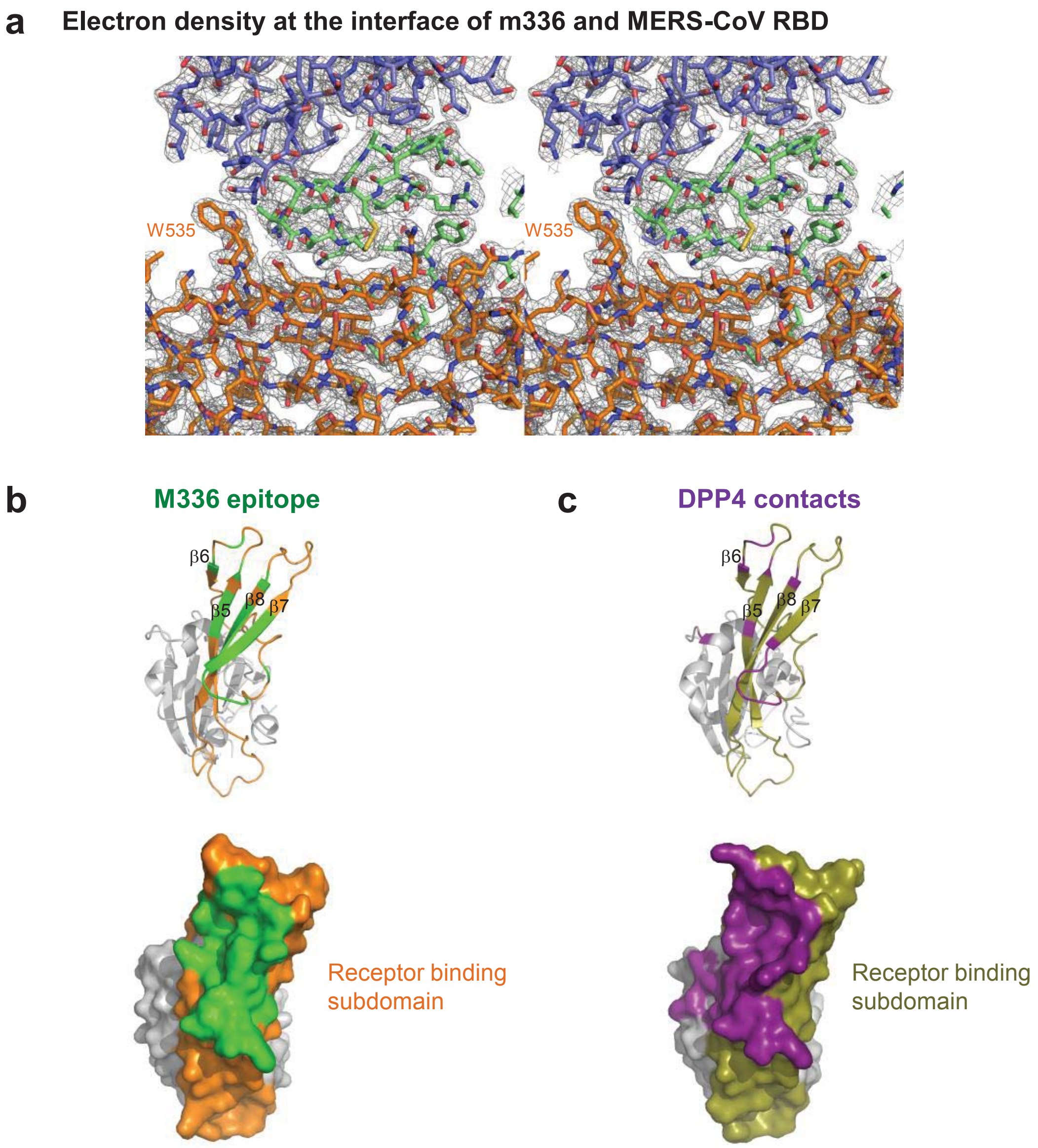

Supplementary Figure 1. Representative electron density map and comparison of m336 epitope and DPP4 binding site on MERS-CoV RBD

(**a**) Stereo view of $2F_o$-F_c electron density at the interface of m336 and MERS-CoV RBD contoured at 1.0 σ level. The MERS-CoV RBD, m336 heavy and light chains are shown with stick representation with Cα atoms colored orange, light green and light blue, respectively. MERS-CoV RBD residue 535 is labeled for view orientation. (**b** and **c**) Side-by-side comparison of m336 epitope and DPP4 binding site on MERS-CoV RBD shown in both cartoon (top) and surface representation (bottom). The epitope of m336 (**b**) is colored in green and the binding site of DPP4 (**c**) is colored in purple on MERS-CoV RBD .

Supplementary Figure 2

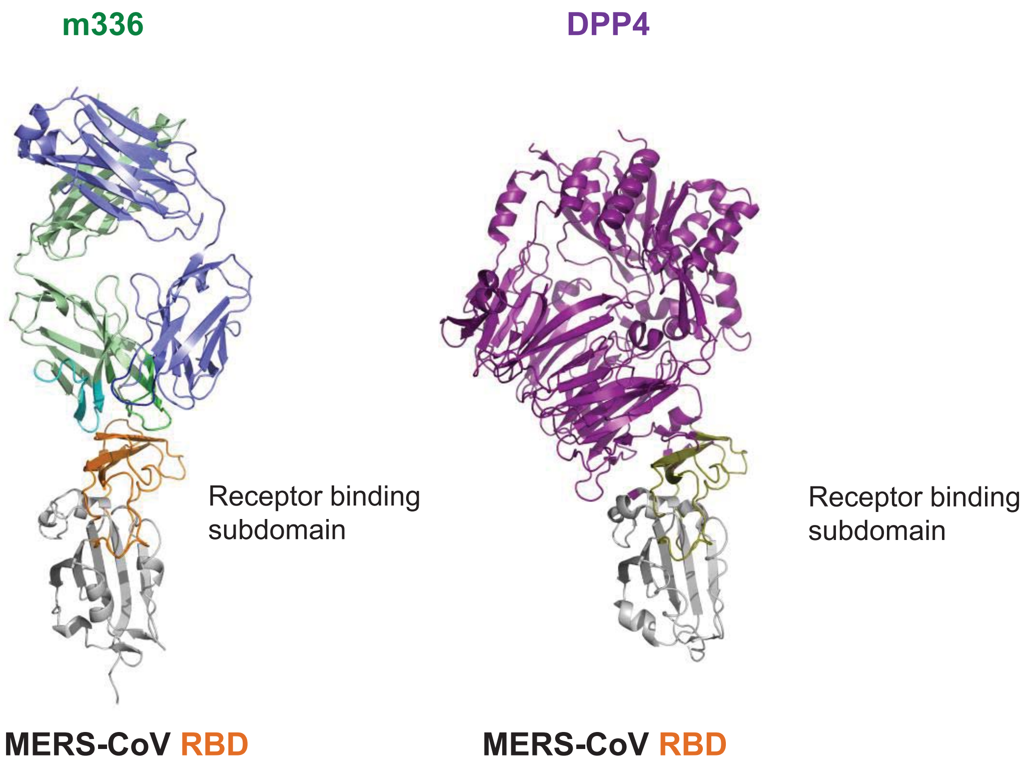

Supplementary Figure 2. m336 mimics receptor DPP4 with similar angle of approach to engage MERS-CoV

The MERS-CoV RBD, m336 heavy and light chains as well as the receptor DDP4 are shown in cartoon representation with the same color scheme as in Figures 1 and 2.

Supplementary Figure 3

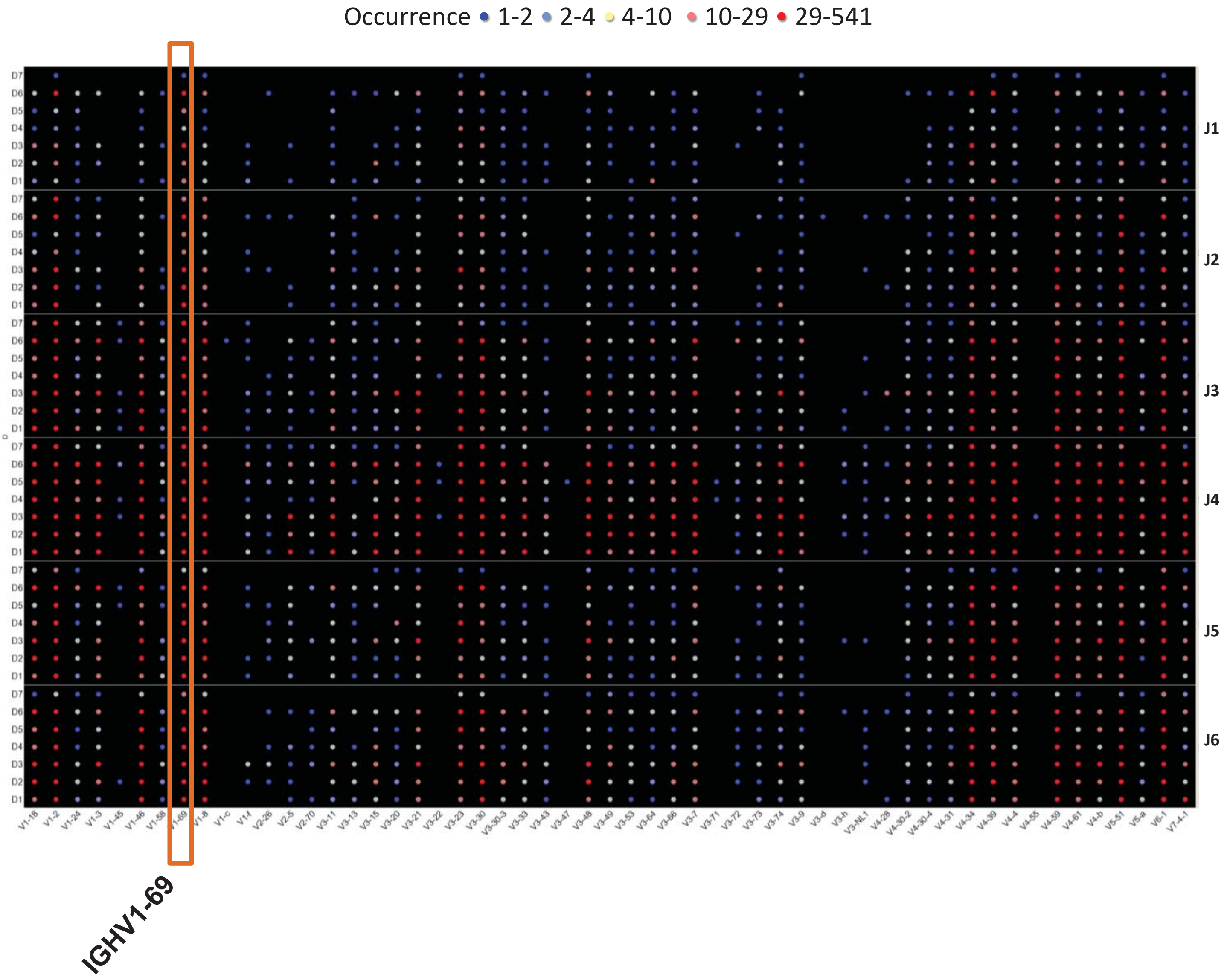

Supplementary Figure 3. VDJ frequencies showing the productive VH rearrangements from different IGHV germline genes as quantified from the 454 sequencing analysis of IgM libraries derived from 69 healthy human subjects
The IGHV1-69 contributes significantly (12.7%) to the antibody repertoires, boxed in orange (out of the total of 74,393 VH sequences).

Supplementary Figure 4

```
                    10         20         30         40         50         60         70           80         90         100
                     |          |          |          |          | a        |          |            |          |          |
KV1-17*01 DIQMTQSPSSLSASVGDRVTITCRASQGIRNDLGWYQQKPGKAPKRLIYAASSLQSGVPSRFSGSGSGTE  FTLTISSLQPEDFATYYCLQHNSYP------------- 95
m336      DIQLTQSPSSLSASVGDRVTITCRASQGIRNDLGWYQQKPGKAPKLLIYAASSLQSGVPSRFSGSGSGTD  FTLTISSLQPEDFATYYCQQLNSYP-LTFGGGTKVEIK 107
SC4098    DIQMTQSPSSLSASVGDRVTITCRASQGIRNDLGWYQQKPGKAPKLLIYAASSLQSGVPSRFSGSGSGTD  FTLTISSLQPEDFATYYCQQLNSYP-PTFGGGTKVEIK 107
AO7R8     DIQMTQSPSSLSASVGDRVTITCRASQSIDSYLNWYQQKPGKPPKLLIYGASGLQSGVPSRFSGSGSGTE  FTLTIRSLQPEDFATYYCLQHNSYP-WTFGQGTKVEVR 107
FLZTE     DIQLTQSPSSLSASVGDRVTITCRASQSISSYLNWYQQKPGKAPKLLIYAASSLQSGVPSRFSGSGSGTE  FTLTISSLQPEDFATYYCLQHNSYP-YTFGQGTKLEIK 107
JEKWS     DIQMTQSPSSLSASVGDRVTITCRASQGISSALNWYQQKTGKAPKLLIYAASSLQSGVPSRFSGSGSGTE  FTLTISSLQPEDFATYYCLQHNSYP-RTFGQGTKLEIK 107
DAP65     DIQMTQSPSSLSASVGDRVTITCRASQSISSYLNWYQQKPGKAPKLLIYAASSLQSGVPSRFSGSGSGTE  FTLTISSLQPEDFATYYCLQHNSYP-LTFGGGTKVEIK 107
DN540     DIQMTQSPSSLSVSVGDRVTITCRASQGIGNELGWFQQKPGRAPKLLIYSASSLGGEVPSRFSGSGSGTE  FTLTISSLQPEDFATYYCLQHNSYP-RTFGQGTKVEIK 107
JRFO1     DIHLTQSPSSLSASVGDRVTITCRASQSISSYLNWYQQKPGKAPKLLIYAASSLQSGVPSRFSGSGSGTE  FTLTISSLQPEDFATYYCLQHNSYP-LTFGGGTKVEIK 107
JE94O     DIQLTQSPSPLSASVGDRVTITCRASQSISSYLNWYQQKPGKAPKLLIYAASSLQSGVPSRFSGSGSGTE  FTLTISSLQPEDFATYYCLQHNSYP-WTFGQGTKVEIK 107
JTG8G     DIQLTQSPSSLSASVGDRVTITCQASQDISNYLNWYQQKPGKAPKLLIYAASSLQSGVPSRFSGSGSGTE  FTLTISSLQPEDFATYYCLQHNSYP-LTFGGGTKVEIK 107
GJIIK     DIQLTQSPSSLSASVGDRVTITCQASQDISNYLNWYQQKPGKAPKLLIYAASTLQSGVPSRFSGSGSGTE  FTLTISSLQPEDFATYYCLQHNSYP-WTFGQGTKVEIK 107
IOLJL     DIQLTQSPSSLSASVGDRVTITCQASQDISNYLNWYQQKPGKAPKLLIYVASSLQSGVPSRFSGSGSGTD  FTLTISSLQPEDFATYYCLQHNSYP-RTFGQGTKVEIK 107
J1LEP     DIQLTQSPSSLSASVGDRVTITCRASQGIRNDLGWYQQKPGKAPKLLIYAASSLQSGVPSRFSGSGSGTD  FTLTISSLQPEDFATYYCLQHNSYP-YTFGQGTKLEIK 107
GW8N9     DIQLTQSPSSLSASVGDRVTITCRASQGIRNDLGWYQQKPGKAPKLLIYAASSLQSGVPSRFSGSGSGTE  FTLTISSLQPDDFATYYCLQHNSYP-WTFGQGTKVEIK 107
H71FM     DIQLTQSPSSLSASVGDRVTITCRASQGISNYLAWYQQKPGKVPKLLIYAASSLQSGVPSRFSGSGSGTD  FTLTISSLQPEDFATYYCLQHNSYP-WTLGQGTKVEIK 107
GLZKI     DIQLTQSPSSLSASVGDRVTITCRASQGISNYLAWYQQKPGKVPKLLIYAASSLQSGVPSRFSGSGSGTE  FTLTISSLQPEDFATYYCLQHNSYP-ITFGQGTRLEIK 107
G0V6M     DIQLTQSPSSLSASVGDRVTITCRASQSISSYLNWYQQKPGKAPKLLIYAASSLQSGVPSRFSGSGSGTE  FTLTISSLQPDDFATYYCLQHNSYP-YTFGQGTKLEIK 107
F8OWF     DIQLTQSPSSLSASVGDRVTITCRASQSISSYLNWYQQKPGKAPKLLIYAASSLQSGVPSRFSGSGSGTE  FTLTISSLQPEDFATYYCLQHNSYP-FTFGQGTRLEIK 107
IIPZV     DIQLTQSPSSLSASVGDRVTITCRASQSISSYLNWYQQKPGKAPKLLIYAASSLQSGVPSRFSGSGSGTE  FTLTISSLQPEDFATYYCLQHNSYP-LTFGGGTKVEIK 107
F2VYW     DIQLTQSPSSLSASVGDRVTITCRASQSISSYLNWYQQKPGKAPKLLIYAASSLQSGVPSRFSGSGSGTE  FTLTISSLQPEDFATYYCLQHNSYP-PTFGGGTKVEIK 107
JOFEJ     DIQLTQSPSSLSASVGDRVTITCRASQSISSYLNWYQQKPGKAPKLLIYAASSLQSGVPSRFSGSGSGTE  FTLTISSLQPEDFATYYCLQHNSYP-PTFGQGTKVEIK 107
JEE23     DIQLTQSPSSLSASVGDRVTITCRASQSISSYLNWYQQKPGKAPKLLIYAASSLQSGVPSRFSGSGSGTE  FTLTISSLQPEDFATYYCLQHNSYP-WTFGQGTKVEIK 107
FRB3E     DIQLTQSPSSLSASVGDRVTITCRASQSISSYLNWYQQKPGKAPKLLIYAASSLQSGVPSRFSGSGSGTE  FTLTISSLQPEDFATYYCLQHNSYP-YTFGQGTKLEIK 107
IVVGA     DIQLTQSPSSLSASVGDRVTITCRASQSISSYLNWYQQKPGKAPKLLIYKASSLESGVPSRFSGSGSGTE  FTLTISSLQPEDFATYYCLQHNSYP-LTFGGGTKVEIK 107
G7S4X     DIQLTQSPSSLSASVGDRVTITCRVSQGISSYLNWYRQKPGKAPKLLIYAASSLQSGVPSRFSGSGSGTE  FTLTISSLQPEDFATYYCLQHNSYP-YTFGQGTKLEIK 107
IZFV5     DIQLTQSPSTLSASVGDRVTITCRASQSISSYLNWYQQKPGKAPKLLIYAASSLQSGVPSRFSGSGSGTE  FTLTISSLQPEDFATYYCLQHNSYP-LTFGGGTKVEIK 107
I0BUK     DVVMTQSPSSLSASVGDRVTITCQASQDISNYLNWYQQKPGKAPKLLIYDASSLESGVPSRFSGSGSGTD  FTLTISSLQPEDFATYYCLQHNSYP-WTFGQGTKVEIK 107
IXGN8     DVVMTQSPSSLSASVGDRVTITCRASQSISSYLNWYQQKPGKAPKLLIYAASSLQSGVPSRFSGSGSGTE  FTLTISSLQPEDFATYYCLQHNSYP-YTFGQGTKLEIK 107
IUQ23     EIVMTQSPSSLSASVGDRVTITCRASQGIRNDLGWYQQKPGKAPKLLIYAASSLQSGVPSRFSGSGSGTE  FTLTISSLQPEDFATYYCLQHNSYP-WTFGQGTKVEIK 107
FG4AV     EIVMTQSPSSLSASVGDRVTITCRASQGISNYLAWYQQKPGKAPKLLIYAASTLQSGVPSRFSGSGSGTE  FTLTISSLQPEDFATYYCLQHNSYP-LTFGGGTKVEIK 107
IAE2T     RHQLTQSPSSLSASVGDRVTITCRASQSIRAYLNWYQQKPGKAPKLLIYAASSLQSGVPSRFSGSGSGTD  FTLTISSLQPEDFATYYCLQHNSYP-LTFGGGTKVEIK 107
GRX7Q     DIQLTQSPSSLSASVGDRVTITCRASQGIRNDLGWYQQKPGKAPKLLIYAASSLQSGVPPRFSGSGSGTD  FTLTISSLQPEDFATYYCQQLNSYP-LTFGGGTKVEIK 107
F42LC     DIQLTQSPSSLSASVGDRVTITCRASQGIRNDLGWYQQKPGKAPKLLIYAASSLQSGVPSRFSGSGSGTE  FTLTISSLQPEDFATYYCQQLNSYP-LTFGGGTKVEIK 107
IG1QK     DIQLTQSPSSLSASVGDRVTITCRASQGIRNDLGWYQQKPGKAPKLLIYAASSLQSGVPSRFSGSGSGTE  FTLTISSLQPEDFATYYCQQLNSYP-RTFGGGTKVEIK 107
IJG1L     DIQLTQSPSSLSASVGDRVTITCRASQGIRNDLGWYQQKPGKAPKLLIYAASTLQSGVPSRFSGSGSGTE  FTLTISSLQPEDFATYYCQQLNSYP-FTFGPGTKVDIK 107
HL0R4     DIQLTQSPSSLSASVGDRVTITCRASQGIRNDLGWYQQKPGKAPKLLIYAASTLQSGVPSRFSGSGSGTE  FTLTISSLQPEDFATYYCQQLNSYP-KTFGGGTKVEIK 107
GQ6EI     DIQLTQSPSSLSASVGDRVTITCRASQGIRNDLGWYQQKPGKAPKRLIYAASSLQSGVPSRFSGSGSGTE  FTLTISSLQPEDFATYYCQQLNSYPPNTFGGGTKVEIK 108
GGESY     DIQLTQSPSSLSASVGDRVTITCRASQGIRNDLGWYQQKPGKAPKRLIYAASSLQSGVPSRFSGSGSGTE  FTLTISSLQPEDFATYYCQQLNSYP-RTFGQGTKVEIK 107
GIA8T     DIQLTQSPSSLSASVGDRVTITCRASQGIRNDLGWYQQKPGKAPKRLIYAASSLQSGVPSRFSGSGSGTE  FTLTISSLQPEDFATYYCQQLNSYP--TFGGGTKVEIK 106
FV24F     DIQLTQSPSSLSASVGDRVTITCRASQGIRNDLGWYQQKPGKVPKLLIYAASTLQSGVPSRFSGSGSGTE  FTLTISSLQPEDFATYYCQQLNSYP-LTFGGGTKVEIK 107
ATEUR     DIQMTQSPSSLSASVGDRVTITCRASQGIRNDLGWYQQKPGKAPKRLISAASTLQGGVPSRFSGSGSGTE  FTLTISSLEPEDFATYYCQQLNSYP-ITFGQGTRLEIK 107
HKAV1     DIQTTQSPSSLSVSVGDRVTIHCRASQDIRNDLAWYQHTPGKAPQRLIYGASKLQSGVSSRFSGSGSGTE  FTLTIFSLQPEDFATYYCQQLNSYPGFTFGPGTKVDIK 108
              *****.**.******** *:.**.*   * *:::..*: *: **  ** * . *..*********:  ***** **:*:******* * ****
```

Supplementary Figure 4. Multiple alignment of m336 V_L-like sequences from naïve IgM libraries derived from 69 healthy human subjects and 2 newborn babies and an anti-rabies virus V_L antibody sequence

Amino acid sequences of 40 V_L antibody domains (denoted by 5-letter codes) that were found similar to that of m336 from 454 sequencing analysis of IgM libraries derived 69 adult s and 2 babies., and a VL antibody chain from anti-rabies virus antibody (SC4098) from the report by Kramer *et al* Eur. J. Immunol. 2005. Red boxes show the pre-existing mutations in the IgM naïve repertoires as identical to that found in m336 V_L suggesting a naturally germline-related m336 V_L.

Supplementary Figure 5

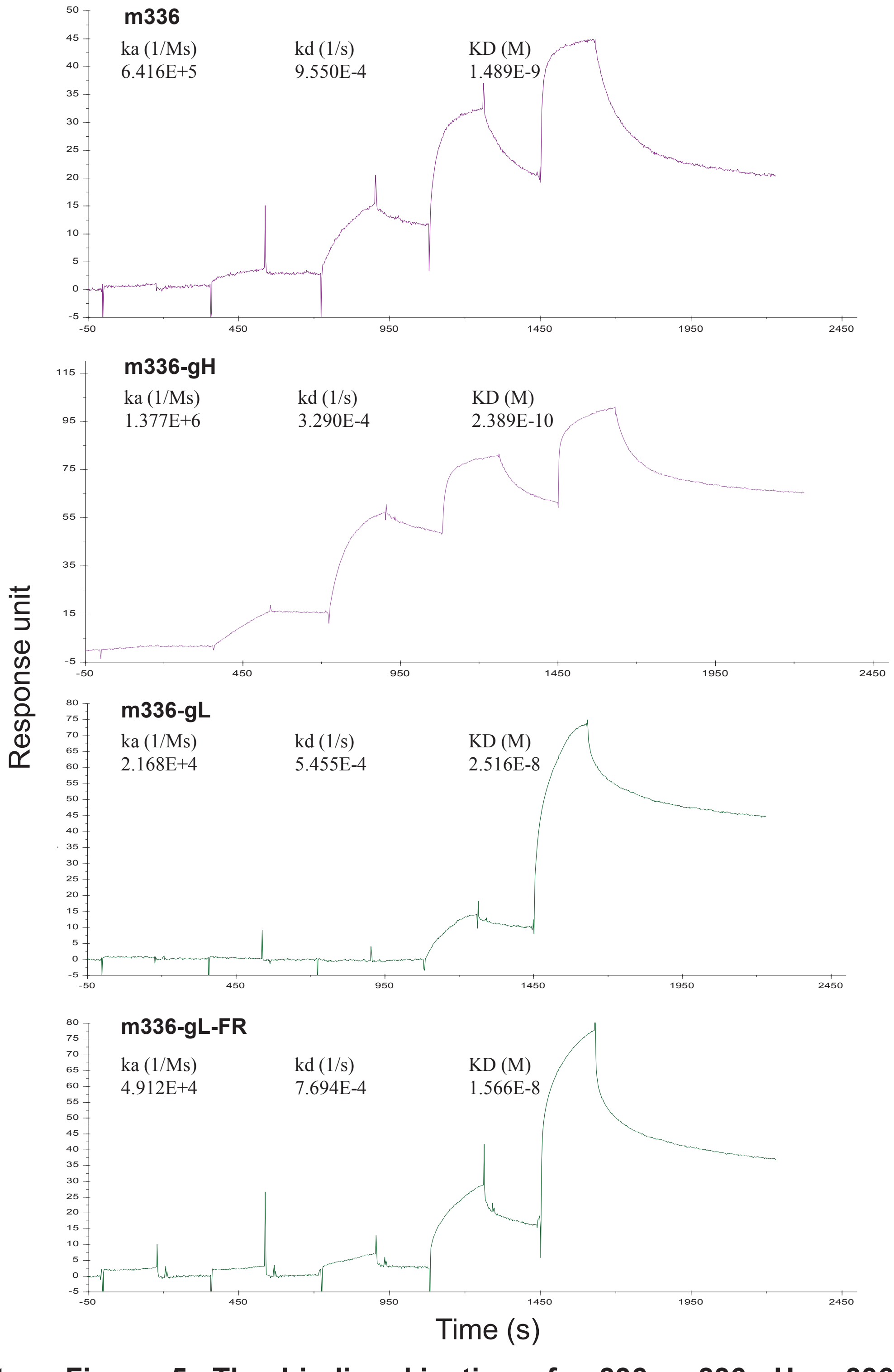

Supplementary Figure 5. The binding kinetics of m336, m336-gH, m336-gL and m336-gL-FR to MERS-CoV S1

Single-cycle surface plasmon resonance analyses were carried out to assess binding of m336 variants to MERS-CoV S1. Antibody at five concentrations were injected incrementally in a single cycle onto a CM5 sensor chip immobilized with MERS-CoV S1 protein. The kinetic profiles are shown in each panel.

Supplementary Figure 6

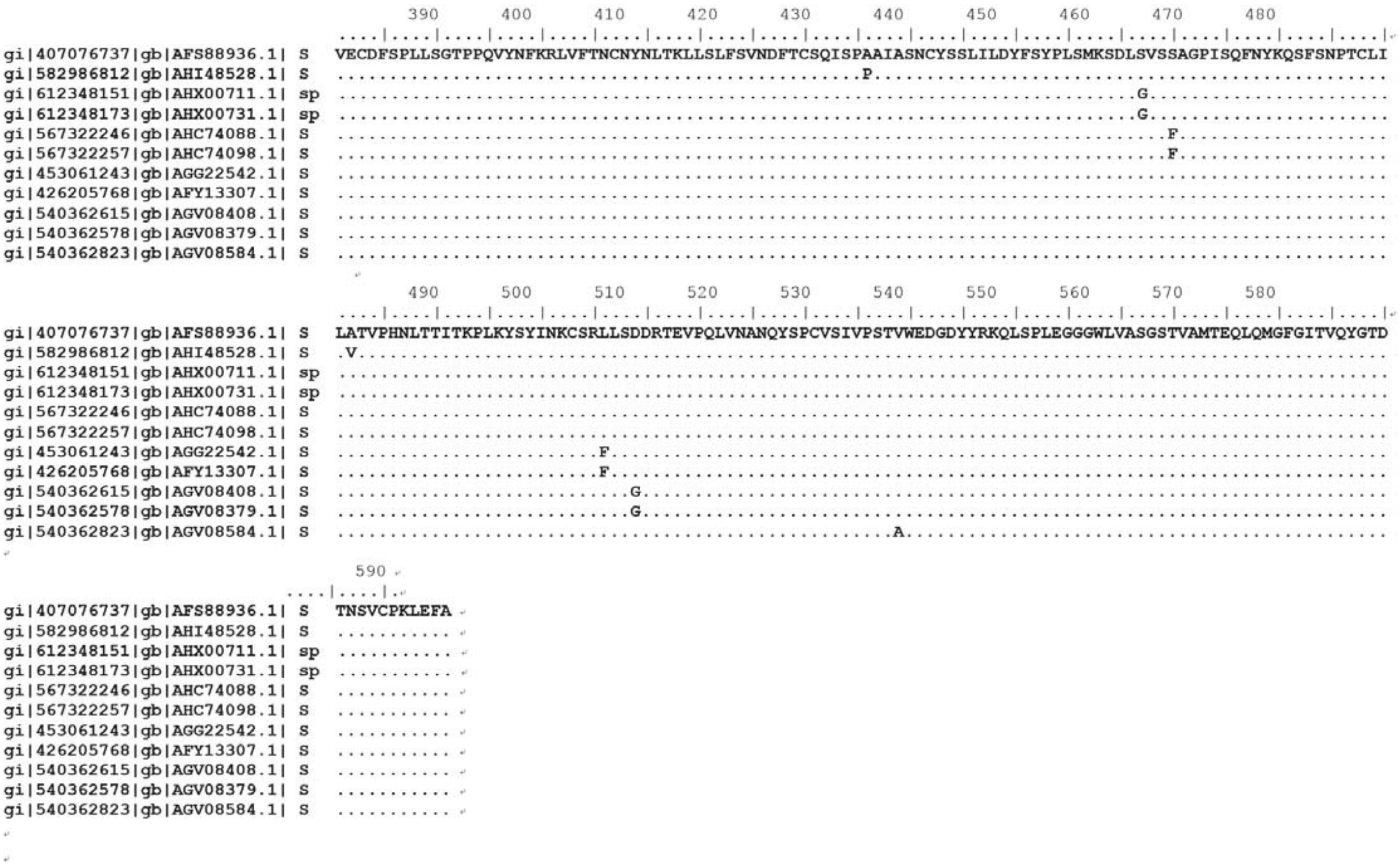

Supplementary Figure 6. Multiple sequence alignment of known MERS-CoV RBD sequences with point mutations from clinical isolates was shown with the prototype EMC/2012 sequence (top)

Isolates harboring the identified A431P, S457G, S460F, A482V, L506F, D509G, and V534A substitution are shown with conserved residues marked as dots.

Supplementary Table 1. Contribution of different m336 regions to its MERS-CoV RBD binding interface

	**Heavy chain (Å^2)**	**Light chain (Å^2)**
**Framework 1**	0	0
**CDR1***	203 (23.6%)	0
**Framework 2**	0	0
**CDR2**	280 (32.6%)	0
**Framework 3**	14 (1.7%)	0
**CDR3**	250 (29.1%)	111 (12.9%)
**Total**	747 (87.1)	111 (12.9%)

*: all complementarity determining regions (CDRs) were defined according to Kabat nomenclature.

Supplementary Table 2. Hydrogen bonds and salt bridges between m336 and MERS-CoV RBD

Hydrogen bonds				
	**m336**	**Distance (Å)**	**MERS-CoV RBD**	**Conserved DPP4 interaction**
1	H:SER30[OG]	3.08	A:ASP510[OD2]	Arg317
2	H:ASN100d[ND2]	3.43	A:GLY538[O]	Gln286
3	H:ARG100e[NH1]	2.75	A:ASP539[OD1]	Lys267
4	H:ARG100e[NH2]	2.98	A:ASP539[OD2]	
5	H:CYS100c[N]	2.70	A:TYR540[O]	
6	H:ASN100d[OD1]	2.96	A:TYR540[N]	
7	H:SER31[O]	2.64	A:TYR540[OH]	
8	H:CYS100c[SG]	3.66	A:ARG542[N]	
9	H:SER31[O]	3.52	A:ARG542[NE]	
10	H:VAL99[O]	3.25	A:ARG542[NH2]	
11	H:SER30[OG]	3.74	A:TRP553[NE1]	
12	L:TYR94[N]	3.14	A:GLU536[OE2]	
13	L:SER93[OG]	2.80	A:TRP535[NE1]	

Salt bridges				
	**m336**	**Distance (Å)**	**MERS-CoV RBD**	**Conserved DPP4 interaction**
1	H:ARG100e[NH1]	2.75	A:ASP539[OD1]	Lys267
2	H:ARG100e[NH2]	2.98	A:ASP539[OD2]	Lys267

Supplementary Table 3. Analysis of the junction showing the mapping of IGHD with a high identity with possible N1/N2 addition along with 3'V- region and 5'J-region.

Analysis of the JUNCTION

D-REGION is in reading frame 2.

Click on mutated (underlined) nucleotide to see the original one:

Input	V name	3'V-REGION	N1	D-REGION	N2	5'J-REGION	J name	D name	Vmut	Dmut	Jmut	Ngc
m336VH	Homsap IGHV1-69*06	tgtgcgagag.	t	tggatattgtagtagtaccagctgc......	aaccgagg	...tgcttttgatatctgg	Homsap IGHJ3*02	Homsap IGHD2-2*03	0	0	0	5/9

Translation of the JUNCTION

Click on mutated (underlined) amino acid to see the original one:

	104	105	106	107	108	109	110	111	111.1	111.2	112.3	112.2	112.1	112	113	114	115	116	117	118	Frame	CDR3-IMGT length	Molecular mass	pI	PhysicoChemical Descriptor (by BRFAA)
	C	A	R	V	G	Y	C	S	S	T	S	C	N	R	G	A	F	D	I	W					
m336VH	tgt	gcg	aga	gtt	gga	tat	tgt	agt	agt	acc	agc	tgc	aac	cga	ggt	gct	ttt	gat	atc	tgg	+	18	2,196.48	7.87	CARVGYCSSTSCNRGAFDIW

Be aware that some allele reference sequences may be incomplete or from cDNAs. In those cases, IMGT/JunctionAnalysis uses automatically the allele *01 for the analysis of the JUNCTION.

Closest D-REGIONs

	Score	Identity
M35648 Homsap IGHD2-2*03 F	118	89.66% (26/29 nt)
J00232 Homsap IGHD2-2*01 F	109	86.21% (25/29 nt)
X97051 Homsap IGHD2-2*02 F	109	86.21% (25/29 nt)

Alignment

```
m336VH                        ttggatattgtagtagtaccagctgcaacc
M35648 Homsap IGHD2-2*03 F    .-----------------------t-tgcc
J00232 Homsap IGHD2-2*01 F    .a----------------------t-tgcc
X97051 Homsap IGHD2-2*02 F    .a----------------------t-tacc
```

IGHD2-2*03 **GYCSSTSC**YA

Supplementary Table 4. Analysis of the nucleotide divergence of m336 heavy chain and light chain from its germline counterpart

Heavy chain:

```
                                     <----------------------------------------------  FR1 - IMGT
                                     1               5                  10                 15
                                      Q   V   Q   L   V   Q   S   G   A       E   V   K   K   P
m336VH                               cag gtg cag ctg gtg cag tct ggg gct ... gag gtg aag aag cct

L22583 Homsap IGHV1-69*06 F          --- --- --- --- --- --- --- --- --- ... --- --- --- --- ---

                                     ------------------------------------------> ________________
                                                     20                 25                 30
                                      G   S   S   V   K   V   S   C   K   A   S   G   G   T   F
m336VH                               ggg tcc tcg gtg aag gtc tcc tgc aag gct tct gga ggc acc ttc

L22583 Homsap IGHV1-69*06 F          --- --- --- --- --- --- --- --- --- --- --- --- --- --- ---

                                     __  CDR1 - IMGT  ______________ <--------------------------
                                                     35                 40                 45
                                                      S   S   Y   A   I   S   W   V   R   Q   A
m336VH                               ... ... ... ... agc agc tat gct atc agc tgg gtg cga cag gcc

L22583 Homsap IGHV1-69*06 F          ... ... ... ... --- --- --- --- --- --- --- --- --- --- ---

                                      FR2 - IMGT  -------------------------> ______________  CDR2
                                                     50                 55                 60
                                      P   G   Q   G   L   E   W   M   G   G   I   I   P   I
m336VH                               cct gga caa ggg ctt gag tgg atg gga ggg atc atc cct atc ...

L22583 Homsap IGHV1-69*06 F          --- --- --- --- --- --- --- --- --- --- --- --- --- --- ...

                                      - IMGT  __________ <----------------------------------------
                                                     65                 70                 75
                                          F   G   T   A   S   Y   A   Q   K   F   Q       G   R
m336VH                               ... ttt ggt aca gca agc tac gca cag aag ttc cag ... ggc aga
                                                          N
L22583 Homsap IGHV1-69*06 F          ... --- --- --- --- -a- --- --- --- --- --- --- ... --- ---

                                     ------------------------------  FR3 - IMGT  ---------------
                                                     80                 85                 90
                                      V   T   I   T   A   D   K   S   T   S   T   A   Y   M   E
m336VH                               gtc acg att acc gcg gac aaa tcc acg agc aca gcc tac atg gag

L22583 Homsap IGHV1-69*06 F          --- --- --- --- --- --- --- --- --- --- --- --- --- --- ---

                                     -------------------------------------------------------> ____
                                                     95                 100            104
                                      L   S   S   L   R   S   E   D   T   A   V   Y   Y   C   A
m336VH                               ctg agc agc ctg aga tct gag gac acg gcc gtg tat tac tgt gcg

L22583 Homsap IGHV1-69*06 F          --- --- --- --- --- --- --- --- --- --- --- --- --- --- ---

                                     __________________________  CDR3 - IMGT  ___________________

                                      R   V   G   Y   C   S   S   T   S   C   N   R   G   A   F
m336VH                               aga gtt gga tat tgt agt agt acc agc tgc aac cga ggt gct ttt
```

```
L22583 Homsap IGHV1-69*06 F          --- -a

                                     _______

                                      D   I   W   G   Q   G   T   M   V   T   V   S   S
m336VH                               gat atc tgg ggc caa ggg aca atg gtc acc gtc tct tca

L22583 Homsap IGHV1-69*06 F
```

Nucleotide (nt) mutations

IMGT labels		V-REGION	FR1-IMGT	CDR1-IMGT	FR2-IMGT	CDR2-IMGT	FR3-IMGT	CDR3-IMGT
Nb of positions including IMGT gaps (nt)		**319** (320)	78	36	51	30	117	7 (8)
Nb of nucleotides		**295** (296)	75	24	51	24	114	7 (8)
Nb of identical nucleotides		**294**	75	24	51	24	113	7
Nb of mutations		**1** (2)	0	0	0	0	1	0 (1)
Mutations	Silent	**0** (1)	0	0	0	0	0	0 (1)
	Nonsilent	**1**	0	0	0	0	1	0
Transitions	a>g	**1**	0	0	0	0	1	0
	g>a	**0**	0	0	0	0	0	0
	c>t	**0**	0	0	0	0	0	0
	t>c	**0**	0	0	0	0	0	0
Transversions	a>c	**0**	0	0	0	0	0	0
	c>a	**0**	0	0	0	0	0	0
	a>t	**0** (1)	0	0	0	0	0	0 (1)
	t>a	**0**	0	0	0	0	0	0
	g>c	**0**	0	0	0	0	0	0
	c>g	**0**	0	0	0	0	0	0
	g>t	**0**	0	0	0	0	0	0
	t>g	**0**	0	0	0	0	0	0

Amino acid (AA) changes

IMGT labels			V-REGION	FR1-IMGT	CDR1-IMGT	FR2-IMGT	CDR2-IMGT	FR3-IMGT	CDR3-IMGT
Nb of positions including IMGT gaps (AA)			**106**	26	12	17	10	39	2
Nb of AA			**98**	25	8	17	8	38	2
Nb of identical AA			**97**	25	8	17	8	37	2
Nb of AA changes			**1**	0	0	0	0	1	0
AA changes	Very similar	(+ + +)	**0**	0	0	0	0	0	0
	Similar	(+ + -)	**0**	0	0	0	0	0	0
		(+ - +)	**0**	0	0	0	0	0	0
	Dissimilar	(+ - -)	**0**	0	0	0	0	0	0
		(- + -)	**0**	0	0	0	0	0	0
		(- - +)	**0**	0	0	0	0	0	0
	Very dissimilar	(- - -)	**1**	0	0	0	0	1	0

Light chain:

```
                                    <--------------------------------------------  FR1 - IMGT
                                    1               5                   10                  15
m336VL                              gac atc cag ttg acc cag tct cca tcc tcc ctg tct gca tct gta
X72808 Homsap IGKV1-17*01 F         --- --- --- a-- --- --- --- --- --- --- --- --- --- --- ---
D88255 Homsap IGKV1-17*02 F         --- --- --- a-- --- --- --- --- --- --- --- --- --- --- ---
M64858 Homsap IGKV1-6*01 F          -c- --- --- a-- --- --- --- --- --- --- --- --- --- --- ---
KM455558 Homsap IGKV1-6*02 F        -c- --- --- a-- --- --- --- --- --- --- --- --- --- --- --t
Z00006 Homsap IGKV1-13*02 F         -c- --- --- --- --- --- --- --- --- --- --- --- --- --- ---

                                    ----------------------------------------->  _______________
                                                    20                  25                  30
m336VL                              gga gac aga gtc acc atc act tgc cgg gca agt cag ggc att ...
X72808 Homsap IGKV1-17*01 F         --- --- --- --- --- --- --- --- --- --- --- --- --- --- ...
D88255 Homsap IGKV1-17*02 F         --- --- --- --- --- --- --- --- --- --- --- --- --- --- ...
M64858 Homsap IGKV1-6*01 F          --- --- --- --- --- --- --- --- --- --- --- --- --- --- ...
KM455558 Homsap IGKV1-6*02 F        --- --- --- --- --- --- --- --- --- --- --- --- --- --- ...
Z00006 Homsap IGKV1-13*02 F         --- --- --- --- --- --- --- --- --- --- --- --- --- --- ...

                                    __  CDR1 - IMGT  ______________  <--------------------------
                                                    35                  40                  45
m336VL                              ... ... ... ... ... aga aat gat tta ggc tgg tat cag cag aaa
X72808 Homsap IGKV1-17*01 F         ... ... ... ... ... --- --- --- --- --- --- --- --- --- ---
D88255 Homsap IGKV1-17*02 F         ... ... ... ... ... --- --- --- --- --- --- --- --- --- ---
M64858 Homsap IGKV1-6*01 F          ... ... ... ... ... --- --- --- --- --- --- --- --- --- ---
KM455558 Homsap IGKV1-6*02 F        ... ... ... ... ... --- --- --- --- --- --- --- --- --- ---
Z00006 Homsap IGKV1-13*02 F         ... ... ... ... ... --c -g- -c- --- -c- --- --- --- --- ---

                                     FR2 - IMGT  ------------------------->  ______________  CDR2
                                                    50                  55                  60
m336VL                              cca ggg aaa gcc cct aag ctc ctg atc tat gct gca ... ... ...
X72808 Homsap IGKV1-17*01 F         --- --- --- --- --- --- -g- --- --- --- --- --- ... ... ...
D88255 Homsap IGKV1-17*02 F         --- --- --- --- --- --- -g- --- --- --- --- --- ... ... ...
M64858 Homsap IGKV1-6*01 F          --- --- --- --- --- --- --- --- --- --- --- --- ... ... ...
KM455558 Homsap IGKV1-6*02 F        --- --- --- --- --- --- --- --- --- --- --- --- ... ... ...
Z00006 Homsap IGKV1-13*02 F         --- --- --- --t --- --- --- --- --- --- -a- --c ... ... ...

                                     - IMGT  __________  <--------------------------------------
                                                    65                  70                  75
m336VL                              ... ... ... ... tcc agt tta caa agt ggg gtc cca ... tca agg
X72808 Homsap IGKV1-17*01 F         ... ... ... ... --- --- --g --- --- --- --- --- ... --- ---
D88255 Homsap IGKV1-17*02 F         ... ... ... ... --- --- --g --- --- --- --- --- ... --- ---
M64858 Homsap IGKV1-6*01 F          ... ... ... ... --- --- --- --- --- --- --- --- ... --- ---
KM455558 Homsap IGKV1-6*02 F        ... ... ... ... --- --- --- --- --- --- --- --- ... --- ---
Z00006 Homsap IGKV1-13*02 F         ... ... ... ... --- --- --g g-- --- --- --- --- ... --- ---

                                    ------------------------------  FR3 - IMGT  ---------------
                                                    80                  85                  90
m336VL                              ttc agc ggc agt gga ... ... tct ggc aca gat ttc act ctc acc
X72808 Homsap IGKV1-17*01 F         --- --- --- --- --- ... ... --- --g --- --a --- --- --- --a
D88255 Homsap IGKV1-17*02 F         --- --- --- --- --- ... ... --- --g --- --a --- --- --- --a
M64858 Homsap IGKV1-6*01 F          --- --- --- --- --- ... ... --- --- --- --- --- --- --- ---
KM455558 Homsap IGKV1-6*02 F        --- --- --- --- --- ... ... --- --- --- --- --- --- --- ---
Z00006 Homsap IGKV1-13*02 F         --- --- --- --- --- ... ... --- --g --- --- --- --- --- ---

                                    -------------------------------------------------------> ____
                                                    95                  100             104
m336VL                              atc agc agc ctg cag cct gaa gat ttt gca act tat tac tgt caa
X72808 Homsap IGKV1-17*01 F         --- --- --- --- --- --- --- --- --- --- --- --- --- --- -t-
```

```
D88255 Homsap IGKV1-17*02 F         --- --- -a- --- --- --- --- --- --- --- --- --- --- --- -t-
M64858 Homsap IGKV1-6*01 F          --- --- --- --- --- --- --- --- --- --- --- --- --- --- -t-
KM455558 Homsap IGKV1-6*02 F        --- --- --- --- --- --- --- --- --- --- --- --- --- --- -t-
Z00006 Homsap IGKV1-13*02 F         --- --- --- --- --- --- --- --- --- --- --- --- --- --- ---

                                    _______  CDR3 -  IMGT _________

m336VL                              cag ctt aat agt tac ccg ctc act ttc ggc gga ggg acc aaa gtg
X72808 Homsap IGKV1-17*01 F         --- -a- --- --- --- --t -c
D88255 Homsap IGKV1-17*02 F         --- -a- --- --- --- --t -c
M64858 Homsap IGKV1-6*01 F          --a ga- t-c -a- --- --t -c
KM455558 Homsap IGKV1-6*02 F        --a ga- t-c -a- --- --t -c
Z00006 Homsap IGKV1-13*02 F         --- t-- --- --- --- --t -a
```

Nucleotide (nt) mutations

IMGT labels		V-REGION	FR1-IMGT	CDR1-IMGT	FR2-IMGT	CDR2-IMGT	FR3-IMGT	CDR3-IMGT
**Nb of positions including IMGT gaps (nt)**		**332** (335)	78	36	51	30	117	20 (23)
**Nb of nucleotides**		**284** (287)	78	18	51	9	108	20 (23)
**Nb of identical nucleotides**		**276** (277)	77	18	50	9	104	18 (19)
**Nb of mutations**		**8** (10)	1	0	1	0	4	2 (4)
**Mutations**	**Silent**	**3** (5)	0	0	0	0	3	0 (2)
	**Nonsilent**	**5**	1	0	1	0	1	2
**Transitions**	**a>g**	**0**	0	0	0	0	0	0
	**g>a**	**1**	0	0	0	0	1	0
	**c>t**	**0** (1)	0	0	0	0	0	0 (1)
	**t>c**	**0**	0	0	0	0	0	0
**Transversions**	**a>c**	**1**	0	0	0	0	1	0
	**c>a**	**0**	0	0	0	0	0	0
	**a>t**	**3**	1	0	0	0	1	1
	**t>a**	**1**	0	0	0	0	0	1
	**g>c**	**1**	0	0	0	0	1	0
	**c>g**	**0**	0	0	0	0	0	0
	**g>t**	**1**	0	0	1	0	0	0
	**t>g**	**0** (1)	0	0	0	0	0	0 (1)

Amino acid (AA) changes

IMGT labels			V-REGION	FR1-IMGT	CDR1-IMGT	FR2-IMGT	CDR2-IMGT	FR3-IMGT	CDR3-IMGT
**Nb of positions including IMGT gaps (AA)**			**110** (111)	26	12	17	10	39	6 (7)
**Nb of AA**			**94** (95)	26	6	17	3	36	6 (7)
**Nb of identical AA**			**89** (90)	25	6	16	3	35	4 (5)
**Nb of AA changes**			**5**	1	0	1	0	1	2
**AA changes**	**Very similar**	**(+ + +)**	**0**	0	0	0	0	0	0
	**Similar**	**(+ + -)**	**1**	1	0	0	0	0	0
		**(+ - +)**	**1**	0	0	0	0	1	0
	**Dissimilar**	**(+ - -)**	**0**	0	0	0	0	0	0
		**(- + -)**	**1**	0	0	1	0	0	0
		**(- - +)**	**0**	0	0	0	0	0	0
	**Very dissimilar**	**(- - -)**	**2**	0	0	0	0	0	2
